# Disrupting the Dok3–Card9 Interaction with Synthetic Peptides Enhances Antifungal Effector Functions of Human Neutrophils

**DOI:** 10.3390/pharmaceutics15071780

**Published:** 2023-06-21

**Authors:** Jia Tong Loh, Joey Kay Hui Teo, Srinivasaraghavan Kannan, Chandra S. Verma, Hong-Hwa Lim, Kong-Peng Lam

**Affiliations:** 1 Singapore Immunology Network, Agency for Science, Technology and Research, 8A Biomedical Grove, Singapore S138648, Singapore; loh_jia_tong@immunol.a-star.edu.sg (J.T.L.); joey_teo@immunol.a-star.edu.sg (J.K.H.T.); lim_hong_hwa@immunol.a-star.edu.sg (H.-H.L.); 2 Bioinformatics Institute, Agency for Science, Technology and Research, 30 Biopolis Street, Singapore S138671, Singapore; raghavk@bii.a-star.edu.sg (S.K.); chandra@bii.a-star.edu.sg (C.S.V.); 3 Department of Biological Sciences, National University of Singapore, 16 Science Drive 4, Singapore S117558, Singapore; 4 School of Biological Sciences, College of Science, Nanyang Technological University, 60 Nanyang Drive, Singapore S637551, Singapore; 5 Department of Microbiology and Immunology, Yong Loo Lin School of Medicine, National University of Singapore, 5 Science Drive 2, Singapore S117545, Singapore

**Keywords:** neutrophils, antifungal immunity, Dok3, Card9, interference peptide

## Abstract

Invasive fungal disease is an emerging and serious public health threat globally. The expanding population of susceptible individuals, together with the rapid emergence of multidrug-resistant fungi pathogens, call for the development of novel therapeutic strategies beyond the limited repertoire of licensed antifungal drugs. Card9 is a critical signaling molecule involved in antifungal defense; we have previously identified Dok3 to be a key negative regulator of Card9 activity in neutrophils. In this study, we identified two synthetic peptides derived from the coiled-coil domain of Card9, which can specifically block Dok3–Card9 binding. We showed that these peptides are cell-permeable, non-toxic, and can enhance antifungal cytokine production and the phagocytosis of human neutrophils upon fungal infection. Collectively, these data provide a proof of concept that disrupting the Dok3–Card9 interaction can boost the antifungal effector functions of neutrophils; they further suggest the potential utility of these peptide inhibitors as an immune-based therapeutic to fight fungal infection.

## 1. Introduction

Fungal infection represents one of the deadliest yet under-recognized infectious diseases in the world. Every year, at least 1.5 million people succumb to severe fungal diseases and the mortality rate continues to rise steadily, catalyzed by the growing population of vulnerable individuals, such as immunocompromised patients undergoing myeloablative chemotherapy and stem cell transplants, as well as a newly emerging group of COVID-19 patients being treated with corticosteroids and tocilizumab [1,2,3]. While antifungal drugs are the mainstream therapies used for fungal diseases, the rapid emergence of drug resistance in the environment due to the widespread use of agricultural fungicides necessitates the development of alternative and diverse antifungal pipelines to reduce the impact of such resistance on the treatment of fungal diseases [4].

The host immune system plays a key role in surveillance and defense against invading fungal pathogens, evidenced by the self-limiting and superficial nature of fungal diseases observed in immunocompetent individuals as opposed to the life-threatening invasive infections affecting immunocompromised patients [5]. During fungal infection, the C-type lectin receptor Dectin-1, expressed on the dendritic cell, macrophage, and neutrophil surfaces, recognizes the β-glucan ligand present on the yeast cell wall and initiates the activation of Card9, a critical adaptor protein involved in antifungal defense [6,7,8]. Card9 consists of an N-terminal CARD domain and a C-terminal coiled-coil domain. Upon activation, the CARD domain of Card9 forms a homophilic interaction with Bcl10, while the coiled-coil domain undergoes ubiquitination by TRIM62 to disrupt its autoinhibition. These processes result in the assembly of a functional Card9-Bcl10-Malt1 (CBM) signalosome, which subsequently turns on downstream NF-kb and MAPK signaling to trigger phagocytosis, as well as the production of antifungal cytokines, such as interleukin (IL)-6, TNFα, and IL-1β for fungal clearance [9,10,11]. Notably, humans with deficiencies or loss-of-function mutations in Card9 are presented clinically with spontaneous fungal infections across multiple sites, including that of the skin, oral cavities, kidneys, and the central nervous system, suggesting its pivotal and indispensable role in mediating antifungal defense [12,13,14,15,16]. As such, interventions that enhance Card9 activity in innate immune cells can potentially boost host antifungal immunity, thereby acting as an immune-based therapy for fungal diseases.

Previously, we have identified a new adaptor molecule, Dok3, to be a critical negative regulator of Card9 signaling in neutrophils. Dok3 contains an N-terminal pleckstrin homology (PH) domain for plasma membrane localization, a central phosphotyrosine binding (PTB) domain to mediate interaction with phosphotyrosine-containing consensus motif, and a C-terminal proline-rich region that serves as a docking site for SH2-containing proteins [17]. Specifically, Dok3 mediates the recruitment of Protein Phosphatase 1 (PP1) to maintain Card9 in its de-phosphorylated and inactive state, thereby dampening downstream antifungal immune responses. Consequently, a loss of Dok3 enhances phagocytosis and antifungal cytokine production via neutrophils, which protect the host from morbidity and mortality induced by a systemic *Candida albicans* infection [18]. Since Dok3 suppresses Card9 activity, disrupting the Dok3–Card9 interaction could potentially be a novel therapeutic strategy used to boost neutrophilic responses to fight fungal infection [17].

In this study, we reported that two synthetic peptides derived from the coiled-coil region of Card9 can specifically interfere with Dok3–Card9 binding to enhance antifungal immune responses in human neutrophils. As such, these peptides represent a novel class of immune-based therapeutics which can act as an adjunctive to current antifungal drugs, thereby enhancing therapeutic efficacy and minimizing the chances of treatment failure due to antifungal resistance.

## 2. Materials and Methods

### 2.1. Peptide Synthesis

**Figure 1:**
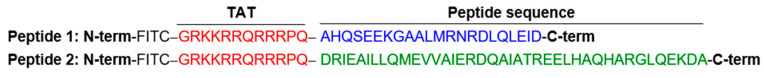
Peptide sequences for disruption of Dok3-Card9 interaction, conjugated to a FITC tag and TAT motif at the N-terminal as indicated.

Peptides were synthesized by Bio Basic Inc. (Singapore) with a 95% purity, as determined by HPLC. The sequences of the peptides are shown in Figure 1.

### 2.2. Isolation of Neutrophils

The venous blood of healthy human donors was collected and neutrophils were purified using an EasySep Direct Human Neutrophil Isolation Kit (Stemcell Technologies, Vancouver, BC, Canada), according to the manufacturer’s protocol. The neutrophils obtained were rested at 37 °C for at least 1 h.

### 2.3. Neutrophil Stimulation

Additionally, 2 × 10^6^ purified neutrophils were preincubated with 20 µg/mL synthetic peptides for 30 min at 37 °C before stimulation with 10 µg/mL zymosan (Invivogen, San Diego, CA, USA), an insoluble preparation of the cell wall from *Saccharomyces cerevisiae*, for 1.5 h (mRNA analysis via qPCR) or 3 h in the presence of GolgiPlug (BD, Franklin Lakes, NJ, USA) (protein analysis via intracellular flow cytometry) at 37 °C in RPMI 1640 media supplemented with 2 mM L-Glutamine, 10% FBS, and penicillin/streptomycin. GolgiPlug is a brefeldin A-containing protein transport inhibitor that blocks intracellular protein transport processes, thereby allowing for the accumulation of cytokines intracellularly for detection by flow cytometry.

### 2.4. Recombinant Vectors

Recombinant vector pcDNA3.1 encoding Card9, Dok3, or the various truncated mutants were synthesized by Bio Basic Inc. (Singapore). Gene sequences for Card9 and Dok3 were extracted from the National Center for Biotechnology Information (NCBI) website. The amino acid position for the PH domain of Dok3 is 9–123, the PTB domain of Dok3 is 157–-215, the CARD domain of Card9 is 10–95, and the coiled-coil domain of Card9 is 127–-396.

### 2.5. Cell Transfection

**Figure 2:**
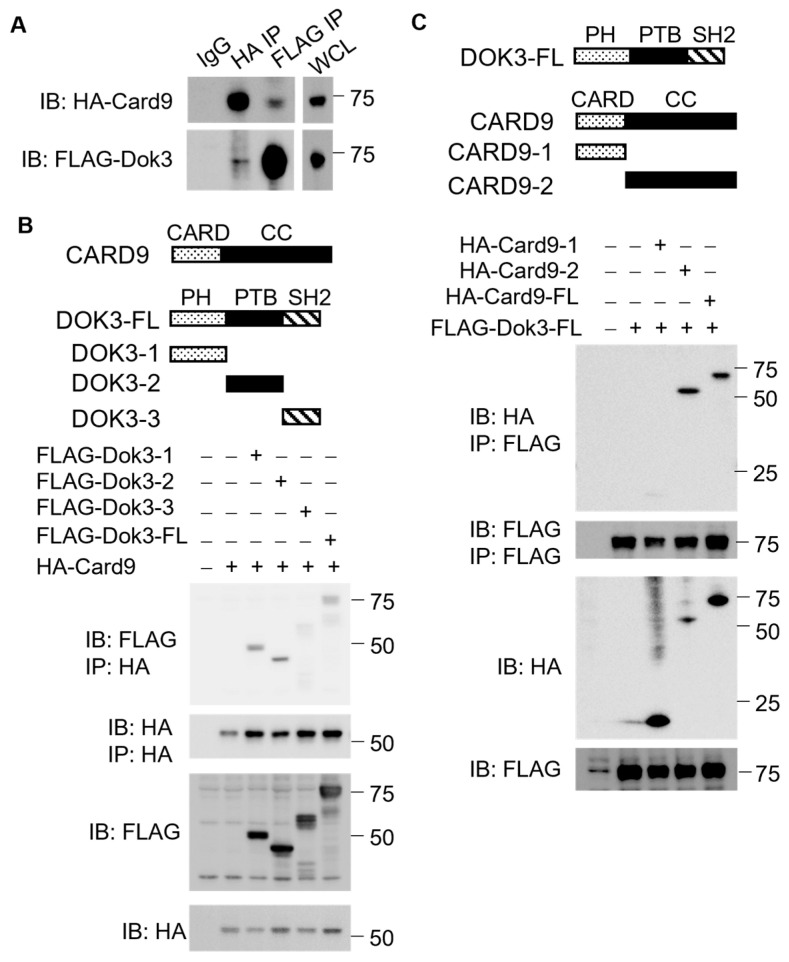
Characterization of the Dok3–Card9 interaction. (**A**) HEK293T cells were transfected with HA-tagged Card9 and FLAG-tagged Dok3. Cell lysates were IP with anti-FLAG or anti-HA antibodies. Precipitates and whole cell lysates (WCL) were immunoblotted with anti-HA and anti-FLAG antibodies. Data shown are representative of three independent experiments. (**B**,**C**) HEK293T cells were transfected with FLAG-tagged Dok3 and HA-tagged Card9 or its truncation variants. Cell lysates were IP with anti-FLAG or anti-HA antibodies. Precipitates and WCL were immunoblotted with anti-HA and anti-FLAG antibodies. Data shown are representative of three independent experiments.

In addition, HEK293T cells were seeded in 6-well plates and transfected the following day with recombinant vectors encoding Card9, Dok3, or various truncated mutants using Lipofectamine 3000 (ThermoFisher Scientific, Singapore); this occurred in the presence or absence of synthetic peptides. Specifically, as shown in Figure 2B, the HEK293T cells were untransfected, transfected with HA-tagged Card9 alone, transfected with HA-tagged Card9 and FLAG-tagged Dok3-1 truncation variant, transfected with HA-tagged Card9 and FLAG-tagged Dok3-2 truncation variant, transfected with HA-tagged Card9 and FLAG-tagged Dok3-3 truncation variant, or transfected with HA-tagged Card9 and full-length (FL) FLAG-tagged Dok3. As displayed in Figure 2C, the HEK293T cells were untransfected, transfected with FLAG-tagged Dok3 alone, transfected with FLAG-tagged Dok3 and HA-tagged Card9-1 truncation variant, transfected with FLAG-tagged Dok3 and HA-tagged Card9-2 truncation variant, or transfected with FLAG-tagged Dok3 and HA-tagged FL Card9. After 24 h of transfection, the cells were harvested by trypsinization and subsequently lysed with a cell lysis buffer (Cell Signaling Technology, Danvers, MA, USA) containing protease and phosphatase inhibitors (Cell Signaling Technology).

### 2.6. Immunoprecipitation (IP) and Western Blotting

The HEK293T cells were lysed with a cell lysis buffer (Cell Signaling Technology) containing protease and phosphatase inhibitors (Cell Signaling Technology) for 1 h at 4 °C with rolling. Cell homogenates were centrifuged at max spin for 5 min at 4 °C; supernatants (whole cell lysate) were collected and IP overnight with anti-FLAG or anti-HA antibodies and pulled down using Protein A/G Plus Agarose beads (Santa Cruz Biotechnology Inc., Dallas, TX, USA) at 4 °C. The beads were washed in lysis buffer 3 times and precipitates were collected by boiling them in SDS sample buffer for 5 min. The whole cell lysates and precipitates were analyzed by Western Blotting, according to standard protocol. Briefly, they were loaded into and electrophoresed in 10% SDS-polyacrylamide gels and subsequently transferred onto polyvinylidene difluoride membranes (Millipore, Burlington, MA, USA). The membranes were blocked with 5% BSA for 1 h at room temperature and probed overnight at 4 °C using the indicated antibodies: anti-FLAG-peroxidase (clone 6F7; Sigma Aldrich, St. Louis, MO, USA) and anti-HA-peroxidase (clone HA-7; Sigma Aldrich) in 1:1000 dilution. SuperSignal West Pico PLUS Chemiluminescent substrate (Thermo Scientific, Singapore) was applied to the blots and the chemiluminescent signals were imaged.

### 2.7. Peptide Uptake Assay

Purified neutrophils were incubated with 1, 5, or 20 µg/mL synthetic peptides for 30 min or 2 h at 37 °C. The uptake of the peptides was measured by flow cytometry, as indicated by levels of FITC fluorescence. 

### 2.8. Cell Death Assay

Purified neutrophils were left untreated or incubated with 1, 5, or 20 µg/mL synthetic peptides for 30 min or 2 h at 37 °C. The cells were stained with APC Annexin V (Catalog 550475; BD Pharmingen, Franklin Lakes, NJ, USA) and Fixable Viability Stain 510 (Catalog 564406; BD Biosciences, Franklin Lakes, NJ, USA), according to the manufacturer’s protocol for identifying apoptotic and dead cells.

### 2.9. Phagocytosis Assay

Heat-killed *Candida albicans* (HKCA) were labeled with calcofluor-white (CFW) (Sigma-Aldrich) for 10 min at room temperature. Subsequently, purified neutrophils were co-cultured with CFW-labeled HKCA at 37 °C for 45 min. Adherent fungal cells were quenched with trypan blue and the cells were washed 5 times with PBS. The extent of the phagocytosis was measured by flow cytometry. 

### 2.10. Flow Cytometry

Neutrophils were surface labeled with fluorochrome-conjugated antibodies for 10 mins at 4 °C in staining buffer (PBS containing 1% BSA). For intracellular staining, cells were fixed and permeabilized using the Cytofix/Cytoperm Kit (BD), according to the manufacturer’s protocol, before staining with the antibodies for 1 h at room temperature. Data were acquired using a LSRII (BD Biosciences) flow cytometer and analyzed using FlowJo software (Tree Star, Mountain View, CA, USA). The following antibodies were used: APC Annexin V (Catalog 550475; BD Pharmingen), Fixable Viability Stain 510 (Catalog 564406; BD Biosciences), and APC/Cyanine7 anti-human TNFα antibody (Clone MAb11; BioLegend, San Diego, CA, USA).

### 2.11. Quantitative PCR

Purified neutrophils were lysed with TRIzol (Gibco, Thermo Fisher Scientific, Billings, MT, USA) and RNA was purified using phenol/chloroform extraction, as per the manufacturer’s protocol. Complementary DNA was reverse transcribed using the RevertAid First Strand cDNA Synthesis Kit (Thermo Fisher Scientific, Waltham, MA, USA), as per the manufacturer’s protocol. The following primers were used for real-time PCR using SYBR Green PCR Master Mix (Applied Biosystems, Waltham, MA, USA):

*Il1b* (forward): AGATGATAAGCCCACTCTACAG;

*Il1b* (reverse): ACATTCAGCACAGGACTCTC;

*Il6* (forward): ACAGCCACTCACCTCTTCAG;

*Il6* (reverse): CCATCTTTTTCAGCCATCTTT;

*hprt1* (forward): GAAAAGGACCCCACGAAGTGT;

*hprt1* (reverse): AGTCAAGGGCATATCCTACAACA.

The mRNA expression of il1b and il6 were normalized to *hprt1* using the 2^−ΔΔCt^ method.

### 2.12. In Silico Model Generation

A structural model of the interaction between the N-terminal pleckstrin homology (PH) and phosphotyrosine binding (PTB) domains of Dok3 with the coiled-coil domain of Card9 was generated using ColabFold [19] via its “advanced interface on Google Colab”. The MMseqs2 method [20] was selected in ColabFold to generate the multiple sequence alignments.

### 2.13. Statistics

Figures and statistical analyses (one-way ANOVA and two-tailed Students’ *t*-test) were generated using the GraphPad Prism software version 9. A *p*-value of less than 0.05 was considered significant.

## 3. Results

### 3.1. Characterization of the Dok3–Card9 Interaction

We have shown previously that Dok3 forms a complex with Card9 endogenously to suppress anti-fungal immunity in neutrophils [17]. To verify if Dok3 interacts directly with Card9, we co-transfected FLAG-Dok3 and HA-Card9 into HEK293T cells. We observed from the co-immunoprecipitation (co-IP) analyses that the anti-HA antibody was able to precipitate FLAG-Dok3; reciprocally, the anti-FLAG antibody can pull down HA-Card9 (Figure 2A). This suggests that Dok3 interacts directly with Card9. Dok3 is a multidomain adaptor protein containing an N-terminal PH, a central PTB, and a C-terminal tyrosine-rich domain. On the other hand, Card9 contains a CARD and a coiled-coil domain. To better understand the molecular interaction between Dok3 and Card9, we further proceeded to map out the specific domain interactions between these two molecules. We examined the physical association of these tagged recombinant proteins in HEK293T cells via co-IP and observed that HA-Card9 can co-IP with full-length Dok3, as well as the variants bearing the PH or PTB domain, but not the SH2 domain, of Dok3 (Figure 2B). On the other hand, FLAG-Dok3 was found to co-purify with full-length Card9 and its variant encoding the coiled-coil, but not CARD, domain (Figure 2C). Together, our data indicate that the PH and PTB domains of Dok3 associate specifically with Card9 through its coiled-coil domain.

### 3.2. Identification of Potential Peptides Which Disrupt Dok3–Card9 Binding

**Figure 3:**
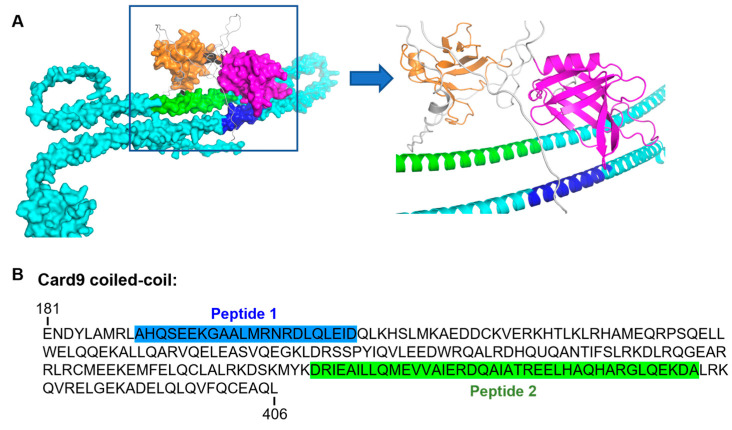
In silico modeling of Dok3–Card9 binding. (**A**) Computational model of Dok3–Card9 complex. Complex between the PH (orange) and PTB (magenta) domains of Dok3 with the coiled-coil domain (cyan) of Card9 is shown as a cartoon/surface. The regions of the coiled-coil domain predicted to interact with Dok3 are highlighted in green and blue and the amino acid sequences are shown in (**B**).

Our earlier study revealed that Dok3 negatively regulates Card9 phosphorylation and activation in neutrophils; consequently, deficiency in Dok3 promotes a “primed” Card9 signaling state which enhances anti-fungal immune responses [17]. As such, we hypothesized that targeting the Dok3–Card9 interaction could represent a potential strategy to boost neutrophilic effector functions to fight fungal infection. To identify potential peptide sequences that can interfere with Dok3–Card9 binding, we first generated a putative complex of the PH and PTB domains of Dok3 interacting with the coiled-coil domain of Card9 via computational modeling. Our in silico model suggested that the PH and PTB domains of Dok3 bind two spatially distinct regions within the coiled-coil domain of Card9 (Figure 3A). Based on their predicted interacting interfaces, we identified two potential peptide sequences (Peptide 1 and Peptide 2) from the coiled-coil region of Card9 that can be used to disrupt the Dok3–Card9 association (Figure 3B).

### 3.3. Synthetic Peptides Are Cell-Permeable and Non-Toxic to Human Neutrophils

**Figure 4:**
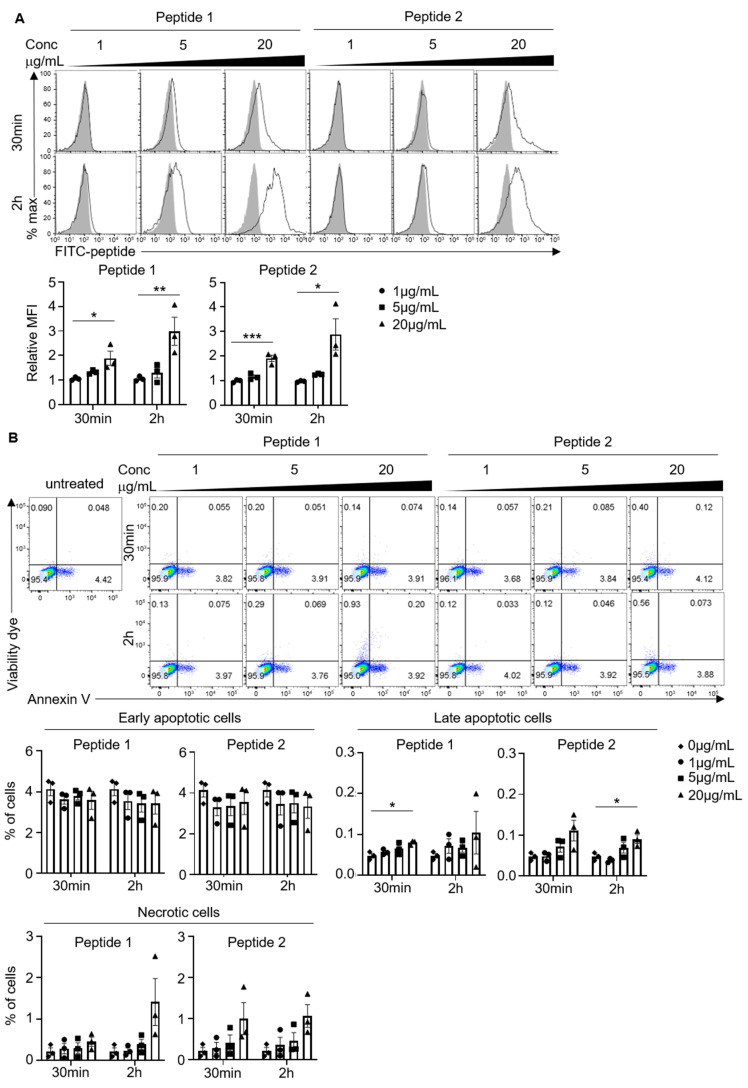
Synthetic peptides are cell−permeable and non−toxic to human neutrophils. Human neutrophils were incubated with indicated concentrations of synthetic peptides for indicated periods of time. (**A**) Uptake of FITC−labeled peptides into neutrophils, as indicated by the relative FITC fluorescence (x−axis) measured by flow cytometry. Filled histograms represent untreated control. Data are shown as mean ± SEM (n = 3, three independent experiments). (**B**) Flow cytometric analyses of early apoptotic (Annexin V^+^ Viability dye^−^), late apoptotic (Annexin V^+^ Viability dye^+^), and necrotic cells (Annexin V^−^ Viability dye^+^). Data are shown as mean ± SEM (n = 3, three independent experiments). * *p* < 0.05, ** *p* < 0.01, *** *p* < 0.001 (one-way ANOVA).

The ability of synthetic peptides to cross the plasma membrane is a prerequisite for them to disrupt Dok3–Card9 binding. As such, we introduced a cell-penetrating human immunodeficiency virus-1 (HIV-1)-derived trans-activating transcriptional activator (TAT) peptide motif (GRKKRRQRRRPQ) at the N-terminal of the synthetic peptides to facilitate membrane penetration and delivery of the synthetic peptides into the cellular environment (Figure 1). We further incorporated a fluorescein isothiocyanate (FITC) tag to visualize the localization of the synthetic peptides (Figure 1). We first evaluated the uptake of individual synthetic peptides in a cell-based assay by incubating purified human neutrophils with increasing concentrations (ranging from 1 to 20 µg/mL) of Peptide 1 or Peptide 2 over a period of 30 min or 2 h. Flow cytometric analyses revealed a dose- and a time-dependent significant increase in the fluorescence of human neutrophils upon the addition of the FITC-labeled synthetic Peptide 1 or 2, indicating that the synthetic peptides can be taken up by and retained in the human neutrophils (Figure 4A).

It has been reported that cell-penetrating peptides may often exhibit cytotoxic effects on cells due to perturbation of membrane integrity, especially at higher concentrations. As such, we evaluated whether the above-tested range of Peptides 1 and 2 can be tolerated by human neutrophils. Here, we identified apoptotic and dead cells using an annexin V probe and live/dead viability stain, respectively. We observed that the percentages of early apoptotic (Annexin V^+^ Viability dye^−^), late apoptotic (Annexin V^+^ Viability dye^+^), and necrotic (Annexin V^−^ Viability dye^+^) neutrophils remain low (less than 5%), even when incubated with peptide concentration as high as 20 µg/mL over a period of 2 h (Figure 4B). Together, these findings suggest that Peptide 1 and Peptide 2 are relatively cell-permeable and non-toxic to human neutrophils. Based on the findings, all subsequent experiments were performed with 20 µg/mL of Peptides 1 and 2 to ensure good bioavailability and minimal cytotoxicity.

### 3.4. Synthetic Peptides Can Disrupt the Dok3–Card9 Interaction

**Figure 5:**
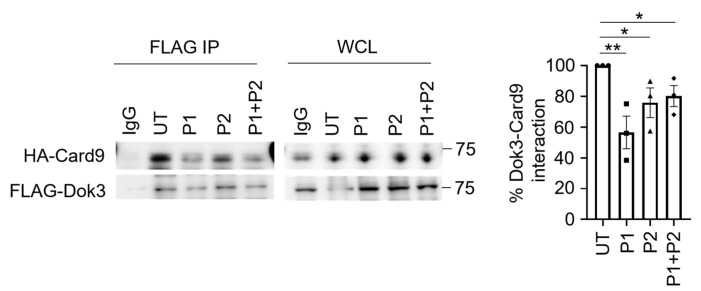
Synthetic peptides can disrupt the Dok3–Card9 interaction. HEK293T cells were transfected with FLAG-tagged Dok3 and HA-tagged Card9. Cells were left untreated (UT) or treated with synthetic peptides. Peptides (P) 1 and 2 were either added individually or in combination with the cells. Cell lysates were IP with anti-FLAG antibodies. Precipitates and WCL were immunoblotted with anti-HA and anti-FLAG antibodies. The data shown are representative of three independent experiments. The bar graph depicts the quantification of the Card9/Dok3 interaction from immunoblots. Data are shown as mean ± SEM (n = 3, three independent experiments). * *p* < 0.05, ** *p* < 0.01 (two-tailed Students’ *t*-test). Circle represents untreated cells. Square represents addition of P1. Triangle represents addition of P2. Diamond represents addition of P1 and 2.

To evaluate the ability of the synthetic peptides in disrupting Dok3–Card9 binding, we co-transfected HA-Card9 and FLAG-Dok3 into HEK293T cells, in the presence or absence of Peptide 1 and/or Peptide 2, and subsequently examined for Dok3/Card9 association via co-IP analyses. An anti-FLAG antibody was able to pull down HA-Card9; the addition of Peptide 1 and/or 2 could significantly reduce the amount of HA-Card9 co-purifying with FLAG-Dok3, with no significant difference observed between Peptide 1 or Peptide 2 or when the peptides were added individually or in combinations (Figure 5). This indicates that the synthetic peptides can specifically disrupt the association between Dok3 and Card9 and that the presence of one peptide is sufficient enough to block this interaction.

### 3.5. Synthetic Peptides Can Enhance Anti-Fungal Immune Responses in Neutrophils

**Figure 6:**
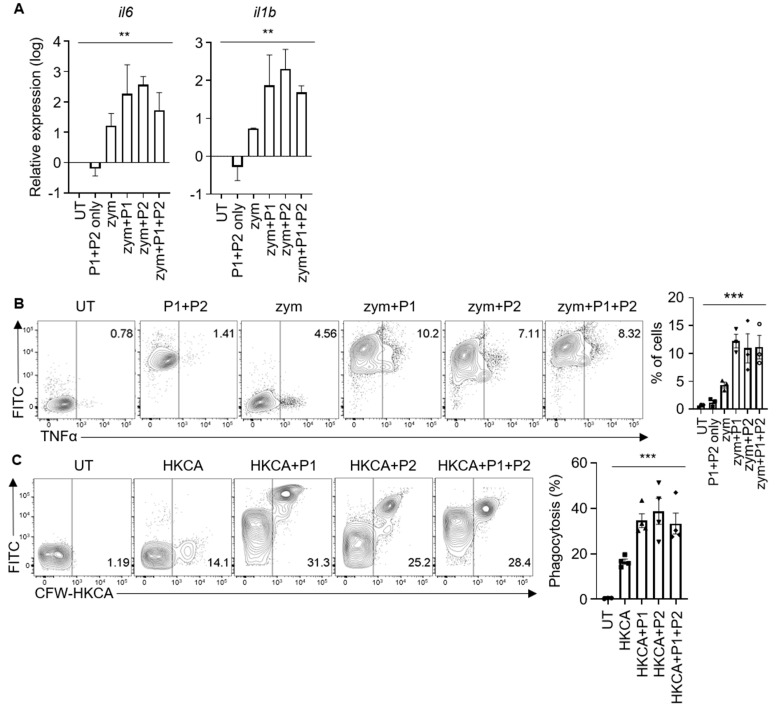
Synthetic peptides enhance the antifungal effector functions of human neutrophils. (**A**) mRNA expression levels of indicated cytokines in human neutrophils. Neutrophils were treated with the following conditions: untreated (UT), Peptide 1 and Peptide 2 alone (P1+P2 only), zymosan alone (zym), or zymosan in the presence of Peptide 1 and/or Peptide 2 (zym+P1 or zym+P2 or zym+P1+P2). Data are shown as mean ± SEM (n = 3, three independent experiments). (**B**) Flow cytometric analyses of TNFα production by human neutrophils. Neutrophils were treated with the same conditions as in (**A**). Graph indicates percentages of TNFα+ neutrophils. Data are shown as mean ± SEM (n = 3, three independent experiments). Circle represents untreated neutrophils. Square represents addition of P1 and 2 only. Triangle represents addition of zymosan. Inverted triangle represents addition of zymosan and P1. Diamond represents addition of zymosan and P2. Open circle represents addition of zymosan, P1 and P2. (**C**) Phagocytosis of CFW−labeled HKCA by human neutrophils. Neutrophils were treated with the following conditions: untreated (UT), HKCA alone (HKCA), or HKCA in the presence of Peptide 1 and/or Peptide 2 (HKCA+P1 or HKCA+P2 or HKCA+P1+P2). Data are shown as mean ± SEM (n = 3, three independent experiments). ** *p* < 0.01, *** *p* < 0.001 (one-way ANOVA). Circle represents untreated neutrophils. Square represents addition of HKCA. Triangle represents addition of HKCA and P1. Inverted triangle represents addition of HKCA and P2. Diamond represents addition of HKCA, P1 and P2.

Next, we determined whether the disruption of the Dok3–Card9 interaction by synthetic peptides can translate into enhanced neutrophilic effector functions. During fungal infection, the recognition of β-glucans and α-mannans on invading fungi by CLRs, such as Dectin-1 and Dectin-2, can initiate the production of pro-inflammatory cytokines by innate immune cells. Here, we stimulated human neutrophils with zymosan, a yeast cell wall component that activates Dectin-1 and TLR2 receptors, and assessed their production of the pro-inflammatory cytokines involved in anti-fungal defense, such as IL-6, IL-1β, and TNFα, in the presence or absence of the synthetic peptides. We observed inductions in mRNA levels of il6 and il1b by human neutrophils following zymosan treatment; these cytokine transcripts were further upregulated in the presence of Peptide 1 and/or Peptide 2 (Figure 6A). Similarly, increased TNFα production was detected in the protein level in response to zymosan stimulation upon the pre-treatment of human neutrophils with the synthetic peptides, with no difference observed between Peptide 1 or Peptide 2 or when the peptides were added individually or in combinations (Figure 6B). Collectively, these findings demonstrate that disrupting the Dok3–Card9 interaction with synthetic peptides can enhance antifungal cytokine production via human neutrophils. Notably, the treatment of the neutrophils with the synthetic peptides alone did not trigger nonspecific cytokine production, suggesting that the concurrent activation of CLR is required for the synthetic peptides to exert their antifungal effects (Figure 6A,B).

Apart from cytokine production, phagocytosis represents another defense mechanism against invading fungal pathogens. To address if disruption of the Dok3–Card9 interaction with synthetic peptides can also promote the phagocytic capacity of human neutrophils, we performed a phagocytic assay with heat-killed C. albicans (HCKA) in yeast form. Here, we observed that pre-treatment with Peptide 1 and/or Peptide 2 can significantly enhance the phagocytic uptake of HKCA by human neutrophils (Figure 6C). Together, our data suggest that the synthetic peptides can boost antifungal effector functions, such as cytokine production and the phagocytosis of human neutrophils; thus, they have the potential to be developed into an effective immunotherapy tool for life-threatening invasive fungal infections.

## 4. Discussion

**Figure 7:**
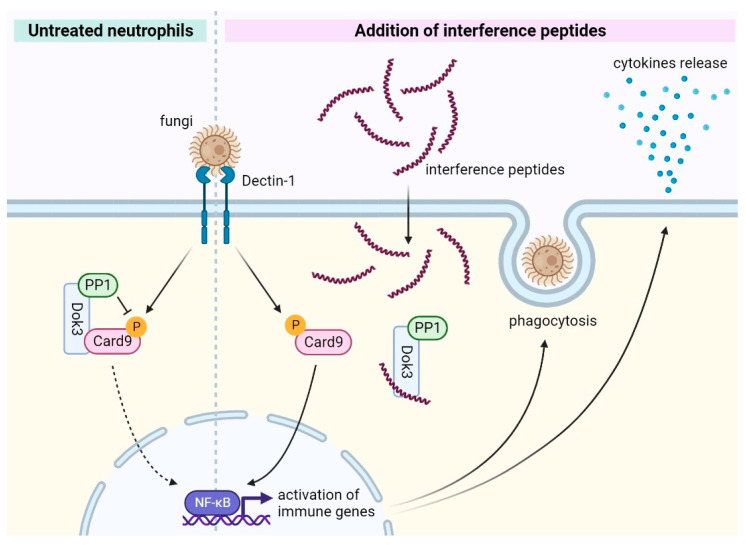
Mechanism for peptide-based interference of the Dok3–Card9 interaction in neutrophils. During the resting state, the Dok3-protein phosphatase 1 (PP1) complex interacts with Card9 to suppress its phosphorylation and activation. In response to fungal infection, the complex will be disrupted and Card9 will be phosphorylated to trigger the downstream activation of NF-kb for phagocytosis and antifungal cytokine production. Synthetic peptides can disrupt the Dok3–Card9 interaction, thereby promoting a “primed” Card9 signaling state which can boost antifungal effector functions to facilitate fungal clearance.

Invasive fungal infection is a serious but neglected public health threat worldwide; the emerging resistance of pathogenic fungi to licensed antifungal drugs highlights the need to expand our therapeutic toolbox against this deadly and destructive disease. In this study, we identified two synthetic peptides which can specifically disrupt the Dok3–Card9 interaction to boost antifungal immunity in human neutrophils (Figure 7). As such, these peptide inhibitors represent a new class of therapeutics aimed at enhancing host innate immunity and demonstrate a novel immune-based approach to antifungal therapy.

Card9 is a key signaling molecule involved in antifungal defense. In response to fungal recognition by C-type lectin receptors, Card9 will be activated to form the CBM complex, which then transduces the signal downstream to initiate immune responses to promote inflammation and fungi killing [13,21]. The importance of this signaling pathway in the antifungal response is demonstrated by human Card9 mutations and deficiencies that manifest spontaneous fungi infections across multiple tissues and organs [12,14,15]. Additionally, amongst the innate immune cells involved in antifungal immunity, neutrophils are largely responsible for the eradication of systemic fungal infections. Previously, we identified that Dok3 is a novel negative regulator of antifungal immunity by recruiting PP1 to de-phosphorylate Card9, thereby dampening downstream immune responses in neutrophils. As such, a loss of Dok3 protects mice from the lethal systemic infection *C. albicans*; this further suggests that disrupting the Dok3–Card9 interaction could potentially remove the brakes on antifungal immunity and enhance neutrophilic effector functions [17]. In this study, based on the predicted interacting interface between the Dok3 PH and PTB domains and the Card9 coiled-coil domain, we derived two synthetic peptides which can specifically disrupt Dok3–Card9 binding. Indeed, these synthetic peptides are able to boost pro-inflammatory cytokine production and the phagocytic capacity of human neutrophils in the presence of fungal-cell-wall-component zymosan, demonstrating their efficacy in fighting fungal infection. Moreover, these synthetic peptides are relatively well-tolerated by neutrophils and do not trigger immune responses in the absence of a fungal ligand, thereby supporting their utility as antifungal therapeutics.

Currently, there are five major classes of antifungal drugs—azoles, echinocandins, polyenes, allylamines, and antimetabolites. They remain the mainstream therapy for invasive fungal infection, even though they are frequently associated with high treatment failure rates owing to the deep-seated nature of fungal infections that makes it difficult for the drugs to reach the affected site, as well as the widespread emergence of antifungal resistance [4]. Despite ongoing efforts to develop new fungicides, the rate of the emergence of drug resistance is higher than the rate of fungicide discovery. Moreover, selection pressure exerted by these chemicals on fungi, in both the environment and clinics, will inadvertently drive the acquisition of resistance over time [22,23]. Hence, the development of alternative treatments for fungal infections is a priority. Since the host immune system plays a critical role in controlling fungal infection, as evidenced by their increased prevalence in patients with underlying immunological dysfunction, one strategy is to utilize immunotherapies to boost the weakened immunity of these high-risk individuals. Indeed, various clinical trials have demonstrated that therapies aimed at restoring the innate immune responses of immunocompromised patients, such as the administration of granulocyte colony-stimulating factors (G-CSF), the administration of granulocyte-macrophage colony-stimulating factors (GM-CSF), or granulocyte transfusion to improve neutrophil numbers in neutropenic patients, can prevent or improve outcomes of fungal infection. Here, we show that synthetic peptides that can interfere with Dok3–Card9 binding can enhance the neutrophil effector functions necessary for fungal clearance from the host. Such immunomodulatory modality can be used as an adjunctive therapy to antifungal drugs, thereby reducing the use of fungicides in clinics to slow down the evolution of fungi resistance.

While this study provides a proof of concept that disrupting the Dok3–Card9 interaction can enhance the antifungal effector functions of neutrophils, future work needs to be carried out to optimize peptide design to facilitate advancement into the clinics. Firstly, even though we showed that Peptide 1 or Peptide 2, when used alone, could boost antifungal immune responses in neutrophils in the presence of fungal ligands, they failed to demonstrate an additive or synergistic effect when added together. According to our in silico model, it is likely that Peptide 1 and Peptide 2 interact with each other since they are located on opposite sides within the coiled-coil domain of Card9; this could possibly reduce their binding efficacy to Dok3. However, more experiments will be required to address this possibility. In addition, one major limitation of the clinical translation of therapeutic peptides is their short half-life in vivo. To enhance the stability and bioavailability of the synthetic peptides, chemical modification of the peptide N- and C-termini or hydrocarbon stapling could be introduced to minimize proteolytic degradation. Moreover, the incorporation of unnatural amino acids has also been reported to have stabilized the synthetic peptides and prolonged their half-lives [24,25]. On the other hand, the binding affinity of the synthetic peptides can be improved via in silico screening to identify hotspots for the Dok3–Card9 interaction. Finally, it will be necessary to conduct preclinical testing of the optimized synthetic peptides in murine models of invasive fungal infections to ascertain their efficacy and safety profile in vivo.

## 5. Conclusions

In conclusion, our study has shown that disrupting the Dok3–Card9 interaction with synthetic peptides in human neutrophils can promote an activated Card9 signaling state, thereby boosting various antifungal effector functions including cytokine production and the phagocytosis of neutrophils. As such, our work provides a promising starting point for the future development of immune-based therapeutics in terms of interference peptides through the enhancement of Card9-dependent antifungal signaling in neutrophils. This can be used as an adjunctive to current antifungal drugs, thereby enhancing therapeutic efficacy and minimizing the chances of treatment failure due to antifungal resistance.

## Data Availability

All data available are contained within the manuscript.
